# Relationship between Negative Running Addiction and Eating Disorder Patterns in Runners

**DOI:** 10.3390/nu13124344

**Published:** 2021-12-01

**Authors:** Montserrat Monserrat Hernández, Ángeles Arjona Garrido, Juan Carlos Checa Olmos, Darío Salguero García

**Affiliations:** Faculty of Humanities, University of Almería, 04120 Almería, Spain; mmh548@inlumine.ual.es (M.M.H.); jcheca@ual.es (J.C.C.O.); dariosalguero@ual.es (D.S.G.)

**Keywords:** runners, eating disorder, negative running addiction, compulsive eating, anorexia nervosa, bulimia nervosa

## Abstract

Current studies show an increase in the risk of eating disorders in runners. Since it is known that abusive exercise can be both a cause and a consequence of such developments, the main objective of the present study was to examine the risk and possible relationships between negative running addiction (NRA), as measured by the reduced and validated SAS-40 scale, and the tendency to be a compulsive eater (measured by YFAS 2.0), anorexia nervosa (AN), and/or bulimia nervosa (BN) (measured by EAT-40). This study highlights the novelty of researching the level of influence of NRA on each defined eating disorder. Method: A total of 167 Spanish-speaking federated runners in cross-country and track running (42% women and 58% men), with an average age of 24 years and an average BMI of 21 kg/m^2^, responded to an online questionnaire that asked about sociodemographic data and the Spanish versions of the SAS-40, YFAS 2, YFAS 3, and YFAS 4. Through a quantitative methodology using logistic regressions—the coefficient of determination and Pearson’s correlation coefficient—we created a sample analysis that related the significant items of the DSM-V to the results of the questionnaires administered, as well as their relationship with the practice of the sport in question and various variables of the environment. Results: The rates of CE, AN, and BN were 65, 11.4, and 16.2%, respectively. The tendency towards CE increased with a lower weight (r = 0.156, *p* < 0.05), not having been overweight in childhood (r = 0.151, *p* < 0.05), and being a long-distance runner (r = 0.123 *p* < 0.05). The risk of AN increased with the absence of menstruation for more than 3 months (r = 0.271 *p* < 0.01), having suffered from childhood obesity (r = 0.213 *p* < 0.05), and being underweight (r = 0.064 *p* < 0.05). The risk of BN increased with having suffered from childhood obesity (r = 0.194 *p* < 0.05), having a higher weight (r = 0.140, *p* < 0.05), and practicing athletics, especially the relay modality (r = 0.044 *p* < 0.05). Conclusions: A considerable number of runners are at risk of suffering from some type of eating disorder. A significant relationship was observed between long-distance runners and the risk of eating disorders (AN, BN, and CE), and the association is stronger for CE than for AN and BN. Lastly, childhood experiences (such as being obese/a healthy weight) were notorious for increasing the risk of eating disorders. Further studies are needed to research each particular parameter and the relationships between the possible levels of dependence on exercise. Level of evidence: Level III, cohort analytic study.

## 1. Introduction

### 1.1. Exercise Dependence in Competitive Athletes

Studies on the benefits of sport suggest that sport is prophylactic for any psychological disorder [1,2]. However, individuals who perform competitive sport may present compulsive exercise behavior that becomes harmful at high levels, such as the feeling of guilt for missed workouts and not reaching set goals, among others. [3,4]. Broadly speaking, a predominance of negative exercise dependence of 0.5 to 3.5% was observed in the general population [3] and was up to more than 50% among competitive athletes [4].

Long-distance runners, in general, are those who need a higher level of motivation compared to those who practice sports for shorter periods, or that require less endurance [5]; this is called super adherence (high commitment to running and high motivation). In this case, commitment to running is shaped in competitive athletes mainly by the number of days they trained for, as well as by calculating the number of kilometers they ran in a week [5]. However, this commitment is sometimes taken to limits that are detrimental to quality of life, leaving social activity, work, and even health aside. This can generate withdrawal syndrome, anxiety, and irritability when training is missed, giving rise to what is called negative running addiction (NRA). NRA is manifested to a greater degree in long-distance runners [6] and competitive athletes due to their greater commitment to training in order to achieve the desired records [6,7]. Initially, NRA was associated mainly with men, but in recent years, studies have found that both sexes experience it [5,6].

Despite being a long-known phenomenon, it is not included in the main diagnostic classification systems (the American Psychiatric Association and the World Health Organization). Even so, exercise addiction shares features with various mental and behavioral disorders that are present in the Diagnostic and Statistical Manual of Mental Disorders, Fifth Edition (DSM-V), such as (1) tolerance, with an increase in the amount of exercise to achieve the desired effect; (2) withdrawal, with negative effects from the absence of exercise, such as irritability, anxiety, and/or sleep problems; (3) lack of control and failure in attempts to reduce or cease exercise practice; (4) intentional effects, with an inability to conform to an established routine; (5) excessive dedication to exercise preparation, performance or regain; (6) the reduction in other social, occupational, and/or recreational activities; (7) the continuation of said practice despite knowing that it causes problems.

It should also be mentioned that there can be two variants of NRA—primary addiction, with the absence of eating disorders; and secondary addiction, in which this factor is included, such as in anorexia nervosa or bulimia nervosa, and where excessive exercise is performed to control energy expenditure [7,8].

### 1.2. Relationship between Negative Running Addiction (NRA) and Anorexia Nervosa (AN) and/or Bulimia Nervoso (BN)

About 400,000 people in Spain suffer from an ED, and it is the third cause of chronic disease in adolescents, according to data from the Spanish Association for the Study of Disorders [7]. Runners are a group at high risk of suffering from this type of disorder [8,9,10].

The relationship between NRA and AN is mainly observed in sports that are especially dependent on thinness and weight [11,12]. This pathology may arise due to two main factors—(1) the high demand for physical and sporting control can lead to this ED [11,13]; (2) people who present a risk or unequivocal manifestations of NRA decide to engage in sports in which thinness is an indispensable requirement (as a way of disguising the disorder) [7,10,11,14,15,16]. It was observed in recent studies that individuals with AN who over-exercise manifest higher levels of anxiety, depression, and perfectionism [17,18].

Other research on the relationship between NRA and compulsive eaters (CE) suggests similarities in the etiology of excessive exercise and attitudes toward disordered eating behaviors [19,20,21,22]. There are also studies reporting that perfectionism and obsessive compulsive disorder cross-sectionally predict compulsive exercise knowledge [16,23].

The WHO determined that the predominance of binge eating disorders is highly observed (up to 30%) in restrictive diets [24]; moreover, it should not be forgotten that competitive runners present diets with these characteristics.

Currently, progressive research on addictive behaviors regarding sports practice and EDs highlights that (1) the predominance of compulsive or excessive exercise in patients with EDs varies from 39 to 45%, and can be as high as 80% in the restrictive subtype of patients hospitalized with anorexia nervosa [22,23]. More specifically, the rate of excessive exercise is 44.4% in patients with AN, 20.6% in those with BN, 20.8% in those with eating disorders not otherwise specified (EDNOS), and in 43.5% of those with a lifetime diagnosis of AN and BN [15]; (2) the predominance of EDs is higher among elite athletes, higher in women than in men, and higher among athletes competing in thinness- and weight-focused sports [11]; (3) compulsive eating is a type of disorder that has a high predominance in endurance sports [11,13]; (4) there is evidence of a relationship between nutrition and mood disorders [25]; (5) the reduction in NRA symptoms (or exercise) is associated with the reduction in EDs [21,26].

The main objective of this study was to analyze the degree to which federated runners have a behavior with a higher risk of developing one of the three most frequent types of eating disorders (ED)—AN, BN, and CE—and, specifically, to observe which variables are the most influential on this manifestation.

## 2. Methods and Data

### 2.1. Participants

For the selection of participants in this study, we chose a sample that was highly representative, as it included 218 professional athletes in any modality of running who train in the Sports Association of Almería (Spain), a local government entity in which non-professional federated runners are promoted and perform under supervision.

The sample error would have been ±3.7% (*p* = *q* = 0.5) for a 95% level of confidence. A total of 167 federated runners from Almería voluntarily completed a questionnaire through a secure online data collection website (Limesurvey). All participants signed the informed consent form.

A total of 97 answers from men and 70 answers from women aged between 18 and 30 years were obtained from this sample (Table 1).

### 2.2. Assessment Instruments

The measurement of sports addiction was performed with the Sports Addiction Scale 40 (SAS-40), which was reduced to 15 items and validated in Spanish [3].

The following five factors were measured: F1—dependence; F2—lack of control, F3—loss of interest in other things; F4—continuity; and F5—concern. The 15 items were distributed in a coherent manner conforming to the typical features of addictive behaviors [7,27].

The tendency towards the development of an ED was measured as follows:

The risk toward compulsive food consumption was measured with the reduced version of the Yale Food Addiction Scale 2.0 (YFAS 2.0) [28]. It was translated into Spanish [29] and the scale applied the Diagnostic and Statistical Manual of Mental Disorders Related to Food Consumption. The threshold for a YFAS 2.0 was reached by passing 2 or more of the 11 criteria set out in the DSM-5, so that the greater the number of matching items, the greater the tendency towards such a pathology.

The tendency or predominance towards AN or BN was measured through the Eating Attitude Test (EAT-40). In this case, the total score allows us to observe behaviors similar to AN and BN, taking as an example the criterion for the analysis of compulsive eaters with the approval of 2 or more of the 11 criteria proposed by the DSM-5 [30].

### 2.3. Procedure

The study was carried out with all the runners federated in the Association of Sports and competing in an athletics modality in the province of Almería (Spain).

Prior to the conduction of the questionnaire, its voluntary, anonymous, and confidential nature was explained to the participants, as well as the content of the research. Subsequently, they were given precise instructions for the completion of the questionnaire. The questionnaire was completed using the Limesurvey application.

### 2.4. Data Analysis

With this sample of runners, using the functions of the SPSS-26 statistical program, we performed basic descriptive analyses, such as the mean, median, minimum and maximum standard deviations.

Additionally, binary logistic regression (stepwise with strategies forward selection) to find the relationship between the binary dependent variables: compulsive eaters (yes/no), anorexia nervosa (yes/no) and bulimia nervosa (yes/no), and the independent variables: sociodemographic: gender, age, BMI, regular menstruation (in women), child obesity (model 1); sports modality (model 2): relay race, medium distance and large distance; and degree of dependence on sports practice (model 3): dependency, lack of control, loss of interest, continuity, and concern.

Pearson’s correlation coefficient and the coefficient of determination to check the degree to which variables (independents) influenced the development of each type of ED (variables depends) studied. The adjusted P value is the significance level to 5% and 1% using the Wald methods.

## 3. Results

Regarding the risk of developing behaviors with risk of tendency towards each type of ED observed, 97.1% of the people surveyed showed a compulsive eating behavior and 65.9% of them had the three highest scores (the development of 9 or more actions out of the 11 considered) (Table 2 and Table 3).

Of the participating runners, 97% also showed a risk of developing anorexia nervosa. Of these, 11.4% scored high (the development of 9 or more of the 11 actions proposed) (Table 2 and Table 3).

As for bulimia nervosa, 100% of them were in the dangerous range of developing the disorder, and a total of 16.2% scored high (the development of 9 or more actions out of the 11 proposed) (Table 2 and Table 3).

We then used Pearson’s correlation coefficients to contrast the common variations of each model analyzed and the scores of the different grouped variables and scales used. Table 4, Table 5 and Table 6 show the correlations obtained.

In relation to the sociodemographic and physical variables (Table 4), it was found that the most influential variable for having a CE disorder with a negative relationship was having a low BMI, followed by not being obese during childhood. In addition, we observed that women with this disorder, unlike AN, tended to have regular menstruation.

When we added the type of running as an independent variable, we observed that being a long-distance runner was the variable that showed the greatest risk of developing CE.

Finally, in analyzing the type of dependence on sports practice, we observed that the level of “F5—concern” (the highest level of NRA) had the strongest relationship and significance, that is, the greater the manifestation of addiction to sport, the greater the risk of developing a CE disorder.

To sum up, with regard to the development of CE, the following statistically significant correlations were obtained: (1) low weight (r = 0.156, *p* < 0.05), (2) not having had obesity in childhood (r = 0.151, *p* < 0.05), (3) long-distance runner (r = 0.123, *p* < 0.05), and (4) high NRA (r = 0.182, *p* = 0.01).

In analyzing AN (Table 5) and taking into consideration the sociodemographic and physical variables, we observed that the most influential factor on the predominance of suffering from this disorder was having suffered from childhood obesity. This was also negatively related to BMI. The absence of menstruation was observed to be a significant consequence of this disorder.

When including the type of run practice as an independent variable in the analysis, we discovered that, as with any of the variables, this disorder could manifest significantly, as it was somewhat more predominant in runners who compete in middle- and long-distance races.

In analyzing the degree of dependence on sports practice, we extensively observed that when runners scored at the F1 level of the NRA scale (level of healthy commitment to practice), it was unlikely that they would manifest the behaviors tending towards this ED. The higher the score on the AN scale (F5 = concern), the more behaviors they developed that were associated with the risk of suffering from AN.

In summary, with respect to the manifestation of risk behaviors for the development of AN, the following statistically significant correlations were obtained: (1) absence of menstruation (r = 0.271, *p* < 0.01), (2) having suffered from childhood obesity (r = 0.213, *p* < 0.05), (3) underweight (r = 0.064, *p* < 0.05), (4) runners (in general, relays: r = 0.007, *p* < 0.05; middle distance: r = 0.026, *p* < 0.05; long distance r = 0.035, *p* < 0.05), and (5) elevated AN (r = 0.236, *p* < 0.01).

When analyzing the behaviors related to the risk of suffering from BN as an ED (Table 6), we observed that, according to the sociodemographic and physical variables, the most significant variable was having suffered from childhood obesity. In addition, BMI had a significant positive relationship with the tendency toward BN.

When we included the type of running as an independent variable, we observed, as with AN, that this ED could be significantly prevalent, and was slightly more common in runners who raced in relays.

Finally, in analyzing the degree of dependence on sports practice, we found that all levels were significantly related to the risk of manifesting BN-related behaviors, with an especially high significance and levels obtained by runners who showed high levels of NRA (F5 = concern).

To sum up, the most significant values for developing behaviors tending towards BN were (1) having suffered from childhood obesity (r = 0.194, *p* < 0.05), (2) high weight (r = 0.140, *p* < 0.05), (3) runners (in general, relays: r = 0.044, *p* < 0.05; middle distance: r = 0.037, *p* < 0.05; long distance: r = 0.023, *p* < 0.05; (4) high NRA (r = 0.191, *p* < 0.01).

## 4. Discussion

The main objective of the present study was to examine the predominance and possible relationships between sports addiction and the risk tendency of developing an eating disorder. In addition, as a novelty, we aimed to research the level of influence of SA on each defined eating disorder (CE, AN, and BN) and to check whether there were independent variables that boosted these developments.

### 4.1. Excessive Physical Exercise as a Risk Factor

The results show that runners are significantly related to the risk of suffering from eating disorders, and those practicing middle- and long-distance races are the most at risk of this phenomenon. It should be taken into consideration that all the runners analyzed were federated competitors. In this regard, numerous studies showed that individuals who practice competitive sports present compulsive cognitions towards exercise and can reach detrimental levels, triggering an NRA [31,32]. This is an important factor in the probability of developing an ED and, in our case, justifies the high similarity of risk behaviors obtained in this study. The reason for this correlation is the idea that excessive body weight limits performance in competition [10,12].

### 4.2. Relationship between the Predominance of ED and NRA

Numerous recent studies support the idea that the predominance of compulsive exercise in patients with AN and BN is used as a means of weight control [32,33,34]. These data may support the idea that people with a tendency to suffer from an ED prefer athletics as a means of justifying their thinness and exercising control over their weight.

Another aspect to highlight is the positive correlation between the development and/or danger of having a CE disorder and a low BMI. This is a new finding since, unlike the indication from the current literature and the DSM-V diagnostic manual (in which the correlation is assumed to be high), this was not the case in the population analyzed (federated runners). The explanation for this phenomenon could be inadequate diet, excessive exercise, stress from competing, or all of these factors combined.

In general, the results obtained in this research show that the higher the level of NRA, the greater the risk of suffering from AN, BN, and/or CE.

### 4.3. Physical and Demographic Characteristics, NRA and ED

No differences were found with respect to age, possibly because the entire sample analyzed was within a small range.

There was also no significance observed with respect to gender. In this case, we did not obtain results similar to those of previous studies that showed a higher prevalence in females [33,35,36]. In our case, this is likely due to the fact that we analyzed runners in which both genders were required to have low-fat body mass and low weight.

Having a high weight in childhood was positively related to a greater development of risk behaviors of presenting AN and BN and negatively related to CE. These data indicate a need for weight control since childhood obesity can develop into obsessive attitudes and behaviors, finally leading to the development of these EDs. On the other hand, the fact that CE disorders do not share these data may indicate that these runners are not concerned about weight, and that the disorder may be more likely to develop due to competitive anxiety or the physiological factors of supercompensation for a deficient nutrient intake.

### 4.4. Sports Modality and ED

As we observed, practicing competitive athletics is considered to be a risk factor for suffering from an ED, and practicing long-distance running scored higher.

In the case of CE, we can consider a physiological cause (beyond the psychological one) for the risk of suffering from this disorder. Practicing long-distance running and being underweight can trigger compulsive food consumption when the nutrition and/or supplementation are inadequate and unsuitable for the phase of training and/or competition in which the runners find themselves.

For AN and BN, the fact that the risk of suffering from these disorders is positively related to having had obesity in childhood offers relevant data on whether the practice of this sport was chosen to maintain low body weight levels. This contrasts with the results of numerous studies that related the practice of excessive physical exercise to the substitution or purging of episodes of overeating [35,37,38]. It is even generally agreed that EDs are not only derived from sporting demands, but that runners with a tendency to suffer from them may choose these sports as a justification for their behavior.

## 5. Conclusions

The predominance of EDs in athletics is a growing problem among those who practice running as a sport. The results of our study also show that the compulsive practice of this sport can increase risk. Therefore, we can confirm that practicing athletics competitively creates a risk of suffering from EDs, with long- and middle-distance running presenting the greatest risk. More specifically, the higher the manifested level of NRA, the greater the risk of AN, BN, or CE.

The present study is consistent with the diagnostic criteria of the pre-DSM-V editions for AN with respect to the presence of amenorrhea. However, this parameter does not necessarily have to be a confirmatory factor for ED; a lack of menstruation can be a consequence of other factors, such as physical or emotional stress or the intake of anovulatory drugs. Therefore, if we analyze the BMI, we can obtain more information, in addition to making a comparison by gender. Taking into account that athletes, in general, have low BMI levels, the tendency of those who are at risk of AN can be a reliable indicator when they have lower levels.

With regard to CE, we considered that it is necessary to continue studying and analyzing the sports population since, as mentioned above, these may be due to both physiological and psychological aspects. In the physiological order, for example, the origin lies in the inadequacy of nutrients and energy.

In view of the abovementioned, we see the need (1) to incorporate a psycho-educational component in the training of runners who are dedicated to competition to address distorted beliefs and cognitions about exercise and nutrition; (2) to abide by the idea of maintaining a regulated training (with its phases of rest and activation), as excessive exercise can trigger injuries, decrease performance, etc.; (3) to include nutritional education and/or nutritionists specializing in this sport; (4) to provide coaches with sufficiently effective tools to detect the danger and/or presence of an ED.

## Figures and Tables

**Table 1:** Basic data of the research screening sample.

	Mean	Standard Deviation	Range
Age	24	2.12	18–30
Hours per week dedicated to run practice	9.2	2.4	4–14
Years practicing running	2.7	1.17	1–10
Body mass index	21.3	3.27	8.59–31.1
	N	%	
Gender			
Female	70	42	
Male	97	58	
Follows alternative diet (vegetarian, vegan, etc.)			
Yes	6	3.6	
No	158	96.3	
Primary discipline practiced			
Short distance	52	31	
Medium distance	23	13.8	
Long distance	36	21.6	
Relay race	17	10.2	
Cross-country running	28	16	
Hurdling	11	6.6	

Source: Own elaboration.

**Table 2:** Questionnaire items related to the criteria found in DSM-5.

Compulsive Eaters	Anorexia Nervosa	Bulimia Nervosa
I have noticed that when I start eating certain meals, I end up eating more than what I had planned. I have noticed that I eat so much that I end up feeling physically unwell, for example, with bloating, stomach pain, nausea, indigestion, etc. I am concerned that I have not been able to avoid consuming certain meals or I have not been able to reduce my consumption. I have noticed that when I am eating certain meals, I have continued to eat them, even though I am not hungry. I have spent a lot of time feeling sluggish, heavy, or tired from overeating. On some occasions when I have eaten certain meals so frequently or in such large quantities, I have spent time wrapped up in negative feelings. I have avoided attending social or work events knowing that certain meals will be available, for fear of overeating. My behavior with respect to food and my way of eating generates discomfort (anxiety, depression, etc.). My food consumption has caused feelings of depression, anxiety, or guilt. Over time, I have noticed that I need to eat more and more to achieve the state of well-being I desire, such as reducing my negative emotions. I have noticed that eating the same amount of food no longer reduces my negative emotions or that it no longer increases the pleasant sensations as it used to do.	I feel like vomiting after meals. I am very afraid of weighing too much. I try not to eat, even if I am hungry. I take into account the calories in the food I eat. I vomit after eating. I worry about my desire to be thinner. I exercise a lot to burn calories. I weigh myself several times a day. I think I burn calories when I exercise. My menstruation comes irregularly. I worry about the idea of having fat on my body.	I am very afraid of weighing too much. I take laxatives. I vomit after eating. I worry about my desire to be thinner. I exercise a lot to burn calories. I weigh myself several times a day. I think about burning calories when I exercise. I purge after an intake that I consider excessive (vomiting, laxative intake, and/or physical exercise). Sometimes I have “binged” on food, feeling that I was unable to stop eating. I feel that food controls my life. I control myself at mealtimes.
All questions have 6 answer options: “never”, “almost never”, “sometimes”, “quite often”, “almost always”, “always”.		

Source: own elaboration.

**Table 3:** DSM-5 scale of runners (%).

	Compulsive Eaters	Anorexia Nervosa	Bulimia Nervosa
0	2.4	-	-
1	0.6	3.0	-
2	-	12.0	3.0
3	-	11.4	6.6
4	-	11.4	14.4
5	3	12.6	10.2
6	6	12.6	18.0
7	9.6	16.2	14.4
8	12.6	9.6	17.4
9	18	7.2	12.6
10	19.8	2.4	3.6
11	28.1	1.8	-

Source: own elaboration.

**Table 4:** Predominance of compulsive eaters according to variables analyzed.

Variables		Model 1	Model 2	Model 3
Socio-demographic and physical	Gender (Female)	0.031	0.051	0.033
	Age	0.076	0.045	0.020
	BMI	−0.156 *	−0.154 *	−0.218 *
	Regular menstruation (Yes)	0.149	0.170	0.198
	Childhood obesity (Yes)	−0.151 *	−0.156 *	−0.145 *
Race type	Relay race		−0.072 *	0.067 *
	Medium distance		0.024 *	0.038 **
	Large distance		0.123 *	0.139 *
Degree of dependence on sports practice	Dependency			−0.042 **
	Lack of control			−0.06 *
	Loss of interest			−0.078 **
	Continuity			0.085 *
	Concern			0.182 **
Coefficient R^2^		0.143	0.261	0.484

* p < 0.001; ** p < 0.005. Source: own elaboration.

**Table 5:** Predominance of anorexia nervosa according to variables analyzed.

Variables		Model 1	Model 2	Model 3
Socio-demographic and physical	Gender (Female)	0.031	0.021	0.118
	Age	0.008	0.025	0.036
	BMI	−0.064 *	−0.059 *	−0.058 *
	Regular menstruation (Yes)	−0.271 **	−0.280 **	−0.288 **
	Childhood obesity (Yes)	0.213 *	0.214 *	0.186 *
Race type	Relay race		0.007 *	0.016 *
	Medium distance		0.026 *	0.032 **
	Long distance		0.035 *	0.039 *
Degree of dependence on sports practice	Dependency			−0.237 **
	Lack of control			0.218 *
	Loss of interest			0.052 **
	Continuity			0.038 *
	Concern			0.236 **
Coefficient R^2^		0.140	0.141	0.363

* p < 0.001; ** p < 0.005. Source: own elaboration.

**Table 6:** Predominance of bulimia nervosa according to variables analyzed.

Variables		Model 1	Model 2	Model 3
Socio-demographic and physical	Gender (Female)	−0.067	−0.080	0.015
	Age	−0.035	−0.032	0.087
	BMI	0.140 *	0.039 *	0.047 *
	Regular menstruation (Yes)	−0.119	−0.121	−0.116
	Childhood obesity (Yes)	0.194 *	0.195	0.173
Race type	Relay race		0.044 *	0.024 *
	Medium distance		0.037 *	0.046 **
	Long distance		0.023 *	0.021 *
Degree of dependence on sports practice	Dependency			0.120 **
	Lack of control			0.127 *
	Loss of interest			0.100 **
	Continuity			0.187 *
	Concern			0.191 **
Coefficient R^2^		0.155	0.157	0.236

* p < 0.001; ** p< 0.005. Source: own elaboration.

## Data Availability

Not applicable.
